# Deep probabilistic traversability with test-time adaptation for uncertainty-aware planetary rover navigation

**DOI:** 10.1038/s41598-026-40109-1

**Published:** 2026-02-18

**Authors:** Masafumi Endo, Tatsunori Taniai, Genya Ishigami

**Affiliations:** 1 Space Robotics Group, Department of Mechanical Engineering, Keio University, Yokohama, 223-8522 Japan; 2 OMRON SINIC X Corporation, Tokyo, 113-0033 Japan

**Keywords:** Planning under uncertainty, Integrated planning and learning, Semantic scene understanding, Space robotics, Engineering, Mathematics and computing

## Abstract

Traversability assessment of deformable terrain is vital for safe rover navigation on planetary surfaces. Machine learning (ML) is a powerful tool for traversability prediction but its inherent predictive uncertainty increases the risk of wheel slips and permanent rover immobilization. To address this issue, we integrate principal approaches to uncertainty handling—quantification, exploitation, and adaptation—into a single learning and planning framework for rover navigation. The key concept is *deep probabilistic traversability*, an end-to-end probabilistic ML model that predicts slip distributions directly from terrain appearance and geometry. This probabilistic model quantifies uncertainties in slip prediction and exploits them as uncertainty-aware traversability costs in path planning. Its end-to-end nature also allows adaptation of pre-trained models with in-situ traverse experience to reduce prediction errors. We perform extensive simulations in synthetic environments posing representative uncertainties in planetary analog terrains. Simulation results show that our method achieves more robust path planning under novel environmental conditions than existing methods.

**Supplementary Information:**

The online version contains supplementary material available at 10.1038/s41598-026-40109-1.

## Introduction

Autonomy is crucial for rapid and extensive robotic planetary exploration due to significant communication delays between Earth and space. In NASA’s Mars 2020 mission, the Perseverance rover’s autonomous navigation system (AutoNav^1^) averaged a traverse distance of 144.4 meters per Martian solar day (24 h 39 min^2^). Although this performance surpasses that of prior rovers^3^, AutoNav has room for improvement in challenging environments, as it primarily achieved rapid traverse on benign terrain. While AutoNav evaluates *traversability* by detecting geometric obstacles on Martian terrain^1^, deformable surfaces covered with fine-grained regolith are more hazardous for rovers than apparent obstacles. These deformable surfaces may induce excessive wheel slip, degrade driving speed, increase energy consumption, and eventually cause permanent entrapment in loose regolith. In one case, NASA’s Opportunity rover spent more than 6 weeks trapped in the rippled sand of the Purgatory Dune, resulting in a prolonged mission timeline^4^. Hence, reliable traversability assessment of wheel slip is essential to ensure the safety of rovers traversing unexplored regions.

Traversability assessment of deformable terrain is challenging because of the complex mechanical interactions arising from rover mobility mechanisms and terrain characteristics, such as mechanical properties and surface geometry^5^. Machine learning (ML) can be used to derive latent relationships between observable environmental features such as terrain appearance and geometry and slip behaviors from training samples. However, despite advances in robot perception and planning^6,7^, no ML model can guarantee perfect predictions owing to inherent *predictive uncertainty*. This uncertainty leads to erroneous predictions, potentially causing the unrecoverable immobilization of rovers operating in planetary environments. Thus, addressing uncertainty in traversability prediction is essential for successful rover navigation.

**Fig. 1:**
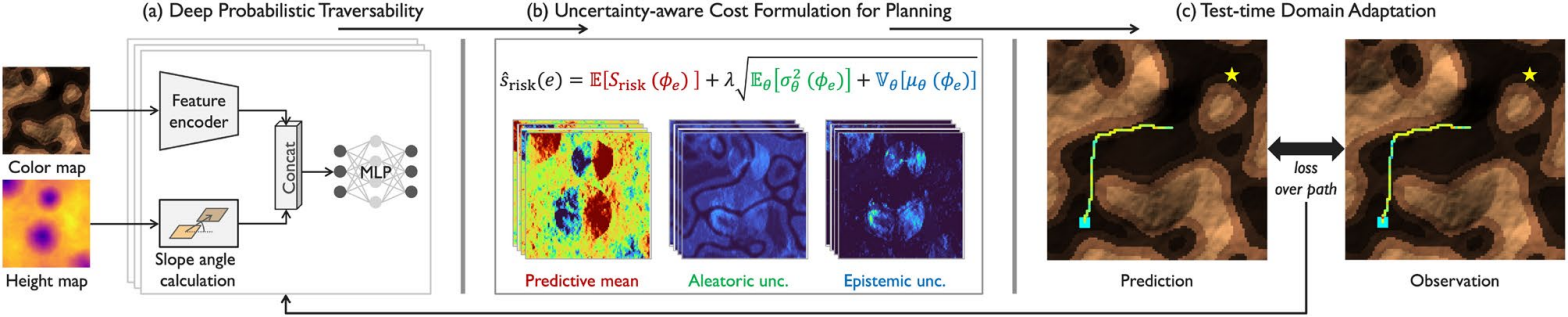
Overview of the proposed unified framework for planetary rover navigation. The framework consists of (**a**) deep probabilistic traversability, (**b**) uncertainty-aware path planning, and (**c**) test-time domain adaptation with in-situ slip measurements, forming an iterative loop to address uncertain traversability prediction.

In ML-based traversability prediction, uncertainty can be managed via three principal approaches: quantification, exploitation, and adaptation. *Quantification* of uncertainty, usually through probabilistic models, provides the degree of uncertainty in the prediction. *Exploitation* of measured uncertainty in planning and decision-making processes helps mitigate the risks of potential prediction errors. *Adaptation* of pre-trained ML models during navigation is also effective in reducing uncertainty owing to limited prior observations. In planetary exploration, ML-based navigation struggles with limited prior traverse experience and uncontrollable environmental factors, such as lighting conditions, which can cause *domain shifts* between training and test environmental conditions.

In this study, we integrate these three uncertainty handling approaches, namely uncertainty quantification and exploitation and model adaptation, into a unified learning and planning framework for safe rover navigation. The key concept in uniting these approaches is *deep probabilistic traversability*, an end-to-end probabilistic ML model using deep neural networks (DNNs) for traversability prediction (Fig. 1a). This model predicts the rover’s wheel slip directly from visual and geometric observations of the target environment while quantifying the predictive uncertainty via probability distributions. Subsequently, this uncertainty can be exploited to reduce the risks associated with imperfect predictions in path planning (Fig. 1b) and is further minimized by adapting the learned distributions to in-situ slip measurements during path execution (Fig. 1c).

We extensively evaluate our method in synthetic planetary environments, focusing on the challenges posed by out-of-domain (OOD) geometry and appearance observations. Comparisons with existing methods demonstrate that our method, with uncertainty-aware planning and domain adaptation, achieves safer rover navigation with fewer immobilizations under novel environmental conditions.

## Related work

ML-based traversability prediction is a key component of off-road autonomy, where *visual* and *geometric* cues are essential. Visual cues capture the diverse geological semantic features that influence terrain traversability. Thus, segmentation-based methods classify symbolic terrain types and identify traversable areas from appearance imagery^8,9^. This appearance-based classification is often combined with geometry-based regression methods to provide further variation in traversability within each class. Classify-then-regress methods model the traversability for each terrain class by correlating the degree of wheel slip with the terrain inclination^10–12^. Mixture-of-experts (MoE) methods further account for terrain class likelihoods for slip regressors^13,14^. Although these combined approaches using visual and geometric cues are effective, they require separate training processes for the classifier and regressor models, which rely on manual annotation.

By using the measured traverse data for supervision, learning traversability from experience has become a viable alternative. This approach provides a direct interpretation of robot–terrain interactions and eliminates the need for labor-intensive manual annotation. Previous studies often used past driving trajectories to auto-generate ground-truth traversability, which assumes scene familiarity with robots^15–17^. Proprioceptive signals, such as force-torque^18^ and linear acceleration^19^, define traversability in continuous-valued regression models. The absence of manual annotation also eases model adaptation through incremental learning with in-situ measurements, improving the robustness to novel situations^20,21^. Our method builds on this learning-from-experience paradigm with an end-to-end DNN backbone, while exploiting both visual and geometric cues.

Off-road navigation algorithms leverage traversability-prediction techniques to ensure robot safety in unstructured environments. In planetary exploration, rover navigation systems adopt classification models to identify traversable regions^22^ and regression models to derive continuous traversability costs from wheel slip^23^. In terrestrial settings, some studies^24–26^ employ traversability models within the model predictive path integral (MPPI) control framework for off-road navigation. However, these methods do not exploit the uncertainty in traversability prediction for safer navigation, as they often rely on deterministic models^24–26^. By contrast, we introduce uncertainty quantification in the prediction models to estimate the reliability of predictions and reduce the risk of rover immobility.

Uncertainty quantification has been extensively studied in ML, including the Bayesian interpretation^27,28^, Monte Carlo dropout^29^, and model ensembles^30^ (see^31^ for details). In robotic decision-making, uncertainty quantification offers valuable tools for risk assessment and is used as a constraint or cost. Several studies have explored chance-constraint formulations for risk-aware off-road navigation^32–34^; however, these constraint-based methods are better suited for Boolean events (e.g., obstacle collision) rather than a range of risks, such as slip. Conversely, cost-based methods estimate conservative traversability by incorporating uncertainty into costs^14,35–40^. This approach involves designing custom-uncertainty-aware traveling costs^36^ or applying statistical risk assessments^40^, such as conditional value-at-risk (CVaR)^41^. Other work explores reinforcement learning approaches that can implicitly exploit uncertainty to learn conservative navigation policies^39^. We employ cost-based uncertainty integration to express a range of risks while allowing for hard-obstacle constraints in regions where rover immobilization is likely to occur.

An alternative to uncertainty avoidance, domain adaptation reduces uncertainties and improves predictions by using new observations in test environments. For planetary exploration, Hedrick et al.^42^ proposed an adaptive navigation system that updates a velocity map online using a Markov random field model. For terrestrial navigation, adaptation using traverse experiences as self-supervision has been studied^20,21^. However, their deterministic predictions without uncertainty modeling may pose serious risks to planetary robots. Kim et al.^43^ presented an active domain adaptation approach for probabilistic traversability but it assumes human monitoring during the adaptation phase before deployment. Our deep probabilistic traversability model adapts *on the fly* to in-situ slip measurements during rover path execution while predicting the occurrence of uncertain and risky regions.

Overall, we focus on planetary rover navigation under the risk of immobility due to uncertain slip on deformable terrain. Our findings quantify, exploit, and reduce the uncertainty in traversability prediction within a unified framework, addressing challenges that have only been partially tackled by existing methods^14,36,40,43–45^.

## Preliminaries

This section outlines a general formulation for path planning and traversability in terms of wheel slip and presents a target map representation for predicting traversability.

### Path planning

The path planning objective is to determine a series of feasible state transitions that allow a robot to navigate toward its destination safely and efficiently. Consider a grid map $\mathscr {G}=(\mathscr {V},\mathscr {E})$ representing an environment in 2D space, where $\mathscr {V}$ is a finite set of nodes *v* enumerating possible robot positions, and $\mathscr {E}$ is a set of edges *e* representing state transitions from each node to its neighbors. We follow a widely used setting, where $\mathscr {G}$ denotes a four-neighbor grid environment with evenly divided grids. Every edge has a strictly positive cost calculated by a travel cost function $f_{\text {cost}}(v,v')$. The optimal path planning problem is formulated to find a path $\mathscr {P}=\left\{ v_1, v_2,..., v_{|\mathscr {P}|}\right\}$, from the start $v_1=v_{\text {start}}\in \mathscr {V}$ to the goal $v_{|\mathscr {P}|}=v_{\text {goal}}\in \mathscr {V}$, with the minimum possible total cost defined as follows:

1 $$\begin{aligned} \min _\mathscr {P} \sum _{i=1}^{|\mathscr {P}|-1}f_{\text {cost}}\left( v_i, v_{i+1}\right) . \end{aligned}$$

### Traversability as wheel slip

Wheeled rovers experience slipping when traversing a deformable terrain. We consider the longitudinal slip ratio *s*^5^ to quantify rover traversability for forward motion ($u_{\text {ref}} \ge 0$) as follows:

2 $$\begin{aligned} {s}={\left\{ \begin{array}{ll} \left( {u}_{\textrm{ref}} - {u}\right) /{u}_{\textrm{ref}},& {u} \le {u}_{\textrm{ref}}: \text {driving state},\\ \left( {u}_{\textrm{ref}} - {u}\right) / {u},& {u}> {u}_{\textrm{ref}}: \text {braking state}, \end{array}\right. } \end{aligned}$$

where *u* and ${u}_{\textrm{ref}}$ denote the actual and reference velocities in the longitudinal direction, respectively. A positive slip ${s \in (0, 1]}$ indicates a traverse slower than the one commanded ($s = 0$ represents no slip), with higher values increasing travel time and, ultimately, causing rover immobilization on deformable terrain. Conversely, a negative slip ${s \in (-1, 0)}$ indicates a traverse faster than the one commanded, which can lead to rover dyscontrol when $s \approx -1$. Although a higher velocity may seem preferable for reducing travel time, it poses operational risks, including deviations from the planned path and potential tip-overs.

### Map representation

Unlike typical path-planning problems, such as shortest pathfinding where edge costs are determined by distance, our formulation for rover traveling requires variable traversability costs to account for slip behavior on deformable terrain. While the actual slip trend is unknown, it primarily depends on terrain geological characteristics, such as mechanical properties and surface geometry in the given environments. Thus, we associate the environment graph with the appearance and geometry of the terrain, particularly terrain surface colors and 3D positions, to predict traversability using ML models. Projecting this information onto graph vertices is feasible for exploration missions, as the HiRISE camera used in Mars exploration captures overhead imagery at 25 cm/pixel with elevation data at 1 m/pixel resolution^46^.

## Uncertainty-aware navigation algorithm

This section presents a unified framework for safer rover navigation that quantifies and exploits uncertainties for planning and further reduces them through test-time adaptation. As formulated above, the framework operates on a four-neighbor grid representation with forward motion, using longitudinal slip as the primary traversability metric. The overall flow of the method is illustrated in Fig. 1 and summarized as follows. Given the appearance and geometry information, we first use the deep probabilistic traversability (DPT) to predict slips and their uncertainties as slip distributions (Fig. 1a). Subsequently, we convert these distributions into traversability costs, accounting for potential prediction errors (Fig. 1b). These costs enable path-planning searches using (1) to determine a path that minimizes the risk of rover immobilization. Finally, we extend this process by incorporating the test-time adaptation of DPT using in-situ slip observations, as well as path replanning (Fig. 1c).

### Deep probabilistic traversability model

We model the DPT using an ensemble of *M* probabilistic DNNs^30^ for robust uncertainty quantification under domain shift. We assume an identical architecture for these DNNs with different parameters $\theta _m$ for each network, denoted as $f_{\theta _m}$. Each network takes a bird’s-eye view environment map of $H \times W$ nodes as the input (Fig. 1a, left). The map represents node-wise RGB colors $I_v \in \mathbb {R}^{3}$ as appearance cues and edge-wise terrain slope angles $\phi _e \in \mathbb {R}$ associated with the rover’s four possible movements at each node as geometric cues. Each network predicts a latent slip distribution for each edge *e* by predicting the mean and variance of the Gaussian distribution $\mathscr {N}(\mu , \sigma ^2)$ as $[\mu _{e,\theta _m}, \sigma ^2_{e,\theta _m}] = f_{\theta _m}(I, \phi _e)$. The DPT then models the slip on each edge *e* as a random variable $S_e$ following a mixture of Gaussian distributions as

3 $$\begin{aligned} S_e {\mathop {=}\limits ^{\Delta }} S_e(\phi _e) \, \sim \, \frac{1}{M} \sum _{m=1}^M \mathscr {N}(s|\mu _{e,\theta _m}, \sigma ^2_{e,\theta _m}). \end{aligned}$$

The networks $f_{\theta _m}$ can accept an arbitrary slope angle $\phi$ to emulate slip distributions $S_e(\phi )$ on edge *e* for hypothetical angles $\phi$ different from the actual angle $\phi _e$. This design is useful for subsequent cost derivations. Thus, $f_{\theta _m}$ is implemented in two steps. First, a convolutional neural network (CNN) extracts a feature map from the color map *I* as $F = \text {CNN}_{\theta _m}(I)$. Subsequently, for each edge $e: v\rightarrow vx2019;$, a multi-layer perceptron (MLP) predicts a Gaussian distribution of slip from the feature pair $(F_v, F_{v'})$ and the slope angle $\phi _e$, as $[\mu _{e,\theta _m}, \sigma ^2_{e,\theta _m}] = \text {MLP}_{\theta _m}(F_v, F_{v'}, \phi _e)$.

Each network $f_{\theta _m}$ starts from independent random initializations and is trained individually to minimize the negative log-likelihood loss:

4 $$\begin{aligned} L_{\theta _m} = -\frac{1}{|\mathscr {E}|}\sum _{e \in \mathscr {E}} \log \left( \mathscr {N}(s_e^*|\mu _{e,\theta _m}, \sigma ^2_{e,\theta _m})\right) , \end{aligned}$$

where $s_e^*$ is the ground-truth slip from training maps.

Note that the Gaussian mixture distribution in (3) has the following forms for the mean and variance:

5a $$\begin{aligned} \mathbb {E}(S_e)&= \mathbb {E}_{\theta }[\mu _{e,\theta }],\end{aligned}$$

5b $$\begin{aligned} \mathbb {V}(S_e)&= \mathbb {E}_{\theta }[\sigma ^2_{e,\theta }] + \mathbb {V}_{\theta }[\mu _{e,\theta }], \end{aligned}$$

where $\mathbb {E}_{\theta }[X_{\theta }] = \frac{1}{M}\sum _{m=1}^M X_{\theta _m}$ denotes the expectation, and $\mathbb {V}_{\theta }[X_{\theta }] = \frac{1}{M}\sum _{m=1}^M (X_{\theta _m}-\mathbb {E}_{\theta }[X_{\theta }])^2$ denotes the variance between the ensemble.

We use the ensemble mean $\mathbb {E}(S_e)$ for slip prediction and the total variance $\mathbb {V}(S_e)$ to quantify uncertainty. In (5b), $\mathbb {V}(S_e)$ is decomposed into two components, expressing different types of uncertainties. The first component, the average of the variance estimates, is known as aleatoric uncertainty and reflects the natural variability from the inherent stochasticity of the observations. The second component, the variance of the mean estimates, is known as the epistemic uncertainty and reflects the gaps between the training and test data.

In the next section, we derive uncertainty-aware traveling costs using these predictions. To simplify the notation, we omit the subscript *e* from $S_e$, $\mu _{e}$ and $\sigma ^2_{e}$ by focusing on the same edge *e*.

### Uncertainty-aware cost formulation

We formulate the cost function $f_{\text {cost}}$ in (1) using the DPT predictions provided in the previous section. The edge cost $f_{\text {cost}}$ is based on the predicted travel time, which encompasses both efficiency and safety since faster traverses lead to shorter travel times with lower slips.

The travel time for each edge $e:v \rightarrow vx2019;$ is given by $t_e = ||\boldsymbol{x}(v)-\boldsymbol{x}(v')||/u(e)$, where $\boldsymbol{x}(v)$ denotes the 3D position at node *v*, and *u*(*e*) denotes the rover velocity during the edge transition. The velocity *u*(*e*) can be calculated from the slip *s* using (2) as follows:

6 $$\begin{aligned} u(e) = {\left\{ \begin{array}{ll} \left( 1-s\right) u_{\text {ref}}, & s \ge 0: \text {driving state},\\ u_{\text {ref}}/\left( 1 + s\right) , & s < 0: \text {braking state}. \end{array}\right. } \end{aligned}$$

The time-based travel costs mitigate the risk of rover immobilization, as these costs approach infinity when complete slip ($s=1$) is predicted. However, they also favor faster rover traverses with larger negative slips ($s \approx -1$), which can endanger the rover. To reduce the risks associated with excessively fast traverse, we adopt the concept of *slip as a risk* introduced in^14^. This concept views any slip state other than that on flat ground ($\phi =0$) as a potential risk. Thus, it evaluates the deviations from this stable state as positive values by inverting the sign of the slip $S(\phi )$ for $\phi < 0$ as follows:

7 $$\begin{aligned} S_{\text {risk}}(\phi ) = {\left\{ \begin{array}{ll} S(\phi ), & \phi \ge 0: \text {ascent},\\ 2\mathbb {E}[S(0)] - S(\phi ), & \phi < 0: \text {descent},\\ \end{array}\right. } \end{aligned}$$

where $S(\phi )$ denotes a random slip variable from (3). This formulation makes edge costs directional, as ascents and descents are treated differently.

Using the slip as a risk, we formulate an uncertainty-aware slip estimate $\hat{s}_\text {risk}$ as follows:

8a $$\begin{aligned} \hat{s}_{\text {risk}}(e)&= \mathbb {E}[S_{\text {risk}}(\phi _e)] + \lambda \sqrt{\operatorname {Var}[S_{\text {risk}}(\phi _e)]}, \end{aligned}$$

8b $$\begin{aligned}&= \mathbb {E}[S_{\text {risk}}(\phi _e)] + \lambda \sqrt{\mathbb {E}_{\theta }[\sigma ^2_{\theta }] + \mathbb {V}_{\theta }[\mu _{\theta }]} . \end{aligned}$$

This estimate computes the expectation of $S_\text {risk}$ using the mixture distribution in (3) and further pessimistically adjusts this risk estimate by adding the standard deviation of $S_\text {risk}$ scaled by the hyperparameter $\lambda \in \mathbb {R}^+$.

After converting $\hat{s}_{\text {risk}}(e)$ to a risk-aware velocity estimate $\hat{u}_{\text {risk}}(e)$ via the slip-to-velocity conversion in (6), we finally derive our cost as a risk-aware travel time as follows:

9 $$\begin{aligned} f_\text {cost}(e:v\rightarrow vx2019;) = \frac{||\boldsymbol{x}(v) - \boldsymbol{x}(v')||}{\hat{u}_{\text {risk}}(e)}. \end{aligned}$$

This cost function evaluates both the traverse efficiency and stability in ascent and descent, while considering the uncertainties in slip predictions.

### Test-time domain adaptation

We incorporate test-time domain adaptation into our framework using actual slip experiences observed in-situ during path execution, as illustrated in Fig. 1c. Specifically, we perform adaptation after each rover movement and re-plan using the updated DPT predictions. This test-time adaptation is enabled by our end-to-end DPT model $f_{\theta _m}$, which is trained solely from slip experience using the loss function in (4), allowing for the backpropagation of the loss computed along the running trajectory.

However, on-the-fly test time adaptation is challenging due to the limited number of available samples. Without sufficient samples, adapting pre-trained models may unintentionally eliminate valuable knowledge acquired during pre-training.

Inspired by previous work on few-shot test-time adaptation^47^, we propose adapting pre-trained models $f_{\theta _m}$ by adding and tuning a fraction of new parameters while keeping all pre-trained parameters $\theta _m$ fixed. Particularly, after every linear and convolutional layer in $f_{\theta _m}$, denoted by $g(\boldsymbol{x}) = W\boldsymbol{x} + \boldsymbol{b}$, we insert a trainable adaptation layer as

10 $$\begin{aligned} \bar{g}(\boldsymbol{x})=\boldsymbol{\gamma } \odot g(\boldsymbol{x}) + \boldsymbol{\beta }, \end{aligned}$$

where $\boldsymbol{\gamma }$ and $\boldsymbol{\beta }$ are vectors of new trainable parameters, and $\odot$ is an element-wise multiplication. We initialize $\boldsymbol{\gamma }$ to $1\!\!1$ and $\boldsymbol{\beta }$ to $0\!\!0$, such that this layer initially preserves the original model behavior, that is, $\bar{g}(\boldsymbol{x}) = g(\boldsymbol{x})$.

Compared with directly updating *W* and $\boldsymbol{b}$ in each layer *g*, this adaptation model better preserves the original semantics of each feature channel in $\boldsymbol{y} = g(\boldsymbol{x})$ by avoiding the mixing of $\boldsymbol{y}$ across the channels. Additionally, having significantly fewer parameters in $\boldsymbol{\gamma }$ than in *W* helps prevent overfitting of the models to a limited number of samples.

## Simulation experiments

In this section, we validate our rover navigation algorithm through extensive simulations to demonstrate its effectiveness under various uncertainties in traversability prediction.

### Dataset

We create a synthetic dataset for path-planning problems, with a focus on the challenge of train–test domain shifts. Thus, we prepare training and validation subsets as in-domain data for training the DPT models and four types of test subsets as out-of-domain data for evaluating navigation performance in unfamiliar environments. Each map instance is a 96 $\times$ 96 grid with a resolution of 1 m per grid cell, providing color and height maps as inputs for navigation and a ground-truth slip map for model training and traverse simulation during testing. Height values are stored as continuous values at each grid cell, with terrain slopes computed from height differences between adjacent cells. The data generation process is as follows:

#### Overall data generation

**Fig. 2:**
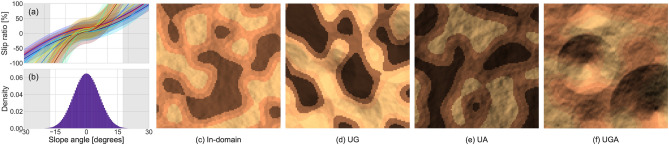
Dataset visualizations. (**a**) Ten latent slip functions associated with terrain classes, with shaded areas indicating the $2\sigma$ ranges of their additive noises. (**b**) Distribution of terrain slope angles (probability density) in the training subset. The gray shading in (**a**) and (**b**) indicates the sampling range of the crater slopes in (**d**) and (**f**). (**c**)–(**f**) Example color maps for the in-domain, unfamiliar geometry, unfamiliar appearance, and unfamiliar geometry and appearance test subsets.

We define 10 terrain classes in the dataset as hidden information. Each class *c* is associated with the nonlinear stochastic slip function $f_c(\phi ) + \epsilon _c$, where $\epsilon _c \sim \mathscr {N}(0, \sigma _c^2)$, as shown in Fig. 2a. The slip function follows $f_c(\phi ) = \pm \alpha _c|\phi |^{\beta _c} + \gamma _c$ (+ for $\phi \ge 0$, − for $\phi < 0$) with class-specific parameters $\alpha _c$, $\beta _c$, and $\gamma _c$ that ensure monotonic increase with $|\phi |$ to capture typical slip behavior. Each class also has an associated appearance color $I_c$ inspired by typical Martian terrain, where we assume each terrain class has a distinct visual signature represented by a unique color. Subsequently, we create *map templates* by generating random rough terrain geometries using fractal terrain modeling^48^. From each map template, we generate multiple map instances by randomly assigning a terrain class to each node or pixel, along with its associated slip function and color. The assignment is performed using random Perlin noise images to form several class clusters on each map. Slip maps are generated by sampling the slips from the assigned slip functions. For the color maps, shading is applied to obtain more realistic observation imagery (Fig. 2c–f). These shaded color maps are computed as $I_v = I_{c(v)} \max (\boldsymbol{n}_v^T \boldsymbol{\ell }, \epsilon )$ for each node *v*, where $\boldsymbol{n}_v$ is a surface normal vector, $\boldsymbol{\ell }$ is a random lighting direction vector, and $\epsilon$ is a small positive constant representing the ambient light level. For the training, validation, and test subsets, 20,000, 5000, and 400 map instances are created from 100, 25, and 40 map templates, respectively.

#### In-domain data generation

We control two design factors for data generation to distinguish between in-domain and out-of-domain (OOD) data. The first factor is the steepness of the geometry in the map templates. The other factor is the lighting angle during shading. Specifically, the light direction vector is sampled in spherical coordinates with an azimuth angle $\psi \sim U(0, 2\pi )$ and a vertical component $z \sim U(z_{\text {min}}, z_{\text {max}})$, where $z_{\text {min}}$ and $z_{\text {max}}$ control the elevation angle range. As in-domain data for training and validation, we adopt fractal terrain geometries, resulting in moderate steepness, with slope angles mostly ranging within $\pm 17^\circ$ (see Fig. 2b). Additionally, we set the lighting parameters $\{z_{\text {min}}, z_{\text {max}}\}$ to $\{0.8, 1.0\}$.

#### Out-of-domain test data generation

For the OOD geometry setting, we introduce steeper crater-like geometries to the fractal-terrain geometries (Fig. 2d,f), with their steepness randomly sampled from the range of $17.5^\circ$–$30^\circ$ (see the highlighted regions in Fig. 2a,b). For the OOD appearance setting, we set the lighting parameters $\{z_{\text {min}}, z_{\text {max}}\}$ to $\{0.3, 0.5\}$. By combining these two OOD factors, we create the following four test subsets:

- In-domain subset (Fig. 2c) simply follows the same data distributions as the training and validation subsets for evaluating baseline performance under familiar environmental conditions.
- Unfamiliar geometry (UG) subset (Fig. 2d) contains OOD crater-like geometries with steeper slopes.
- Unfamiliar appearance (UA) subset (Fig. 2e) contains OOD appearances with darker shading due to lower lighting angles.
- Unfamiliar geometry and appearance (UGA) subset (Fig. 2f) combines both OOD factors.

Each test subset contains 100 map instances generated from 10 map templates. Also, we prepare a smaller dataset of 40 maps, similar to the test subsets, for tuning the hyperparameters of planning and adaptation algorithms.

One could consider using real-world data directly; however, real-world data often remain constrained in variety, potentially resulting in biased verification or mere demonstration of the proposed approach. By contrast, the datasets introduced in this section are more extensive, covering a wide range of scenarios, including OOD cases. As a result, these datasets enable more thorough evaluations compared to using a single real-world dataset.

### Implementation details

For DPT, we use $M=10$ networks. Each network $f_{\theta _m}$ adopts U-Net^49^ with pretrained ResNet-18^50^ as the encoder. We also apply vector embedding that projects slope angles into high-dimensional space for rich feature representation and balanced fusion with color features in the MLP. Here, the MLP consists of three fully connected layers with ReLU^51^ activations and outputs the mean and variance of the slip distribution. During pre-training, each network $f_{\theta _m}$ is trained on the training subset for 100 epochs using the Adam optimizer^52^ with a learning rate of $10^{-3}$ and batch size of 256. For each network, the model weights that produce the lowest validation loss are used for evaluating on the test subsets. Planning is performed using an A* search^53^, which provides an optimal solution for grid maps if one exists. No solution exists if high-slip regions $(s=1)$ disconnect the start and goal. Both planning and execution assume a velocity $u_{\text {ref}}$ of 0.1 m/s. In each adaptation step, backpropagation runs for 10 iterations with a learning rate of $10^{-3}$ for each $f_{\theta _m}$ to exploit the lowest-loss models for subsequent replanning.

**Table 1:** Quantitative path planning results on 400 problem instances from the four test subsets.

	GT	In-domain			UG			UA			UGA		
	Cls	Suc$\uparrow$	$T_{\text {total}}\downarrow$	$s_{\text {max}}\downarrow$	Suc$\uparrow$	$T_{\text {total}}\downarrow$	$s_{\text {max}}\downarrow$	Suc$\uparrow$	$T_{\text {total}}\downarrow$	$s_{\text {max}}\downarrow$	Suc$\uparrow$	$T_{\text {total}}\downarrow$	$s_{\text {max}}\downarrow$
Seg+EV^10^	$\checkmark$	**99**	**24.1**±0.9	48.5±13.3	93	**24.9**±1.5	61.1±17.6	84	**25.1**±1.1	64.4±19.7	66	29.8±34.6	77.2±20.9
MoE+CVaR^14^	$\checkmark$	**99**	24.4±1.1	**45.6**±12.2	**94**	25.1±1.4	57.2±16.8	87	25.5±1.4	65.7±18.5	69	**26.2**±1.9	77.6±18.8
**Ours**		98	24.6±1.2	46.7±12.9	93	25.5±1.5	**56.4**±14.0	98	25.2±1.1	57.2±12.9	87	26.4±2.5	61.8±18.4
Ours w/DA		98	24.6±1.3	47.9±13.3	91	25.4±1.6	56.6±15.8	**99**	25.2±1.4	**55.2**±13.9	**90**	26.5±3.0	**58.6**±17.1

Suc, $T_{\text {total}}$, and $s_{\text {max}}$ denote success rate [%], total time [min], and maximum slip [%]. Suc and $s_{\text {max}}$ indicate path safety, and $T_{\text {total}}$ reflects path efficiency. $T_{\text {total}}$ and $s_{\text {max}}$ are shown as mean ± standard deviation. GT indicates the use of ground-truth class (Cls) annotations.

### Experimental setup

For each instance, the planners search for a path from the start (16 m, 16 m) to the goal (80 m, 80 m). After finding a solution, the rover navigates the map while receiving noisy edgewise slips $s_e$. A solution is considered successful if the rover never observes $s_e \ge$ 1 (rover immobilization) or $s_e \le -1$ (dyscontrol situation) along the path toward the goal. We employ the following three performance metrics:

- Success rate (Suc) [%] denotes $100 \times N_{\textrm{success}}/N_{\textrm{total}}$ where $N_{\textrm{total}}$ is the total number of problem instances and $N_{\textrm{success}}$ is the number of successful path executions.
- Total time ($T_{\text {total}}$) [min] measures the path efficiency as total traversing time per successful path.
- Maximum slip ($s_{\text {max}}$) [%] evaluates path safety by calculating $100 \times \max (\boldsymbol{s_e})$ post-execution, including failure cases. $\boldsymbol{s_e}$ denotes the experienced slips; a lower $s_{\text {max}}$ indicates a safer traverse.

We run our method using $\lambda =2.0$ in (8) with and without domain adaptation (DA). We compare different prediction models and risk evaluation methods to validate the proposed approach. As a baseline, we adopt a segmentation-based planner (Seg+EV)^10^ that selects single class-associated Gaussian processes based on terrain classification and simply calculates the expected values (EVs) of the GPs for planning costs, ignoring prediction uncertainties. We also evaluate a MoE-based risk-aware planner (MoE+CVaR)^14^, which integrates multiple GPs with terrain classification likelihoods and calculates risk-aware planning costs with CVaR using $\alpha =0.9$. These methods use the same CNN architecture as ours for terrain classification. However, their training of CNNs and class-associated GPs relies on additional ground-truth (GT) class annotations from the training subset (see GT in Table 1).

### Results

#### Quantitative comparison

Table 1 summarizes the path-planning performance across the four test subsets. Overall, our approach performs comparably to the baseline methods for the in-domain and UG subsets and significantly outperforms them for the UA and UGA subsets, despite using only slip annotations for training. Comparisons of our results with each baseline and the impact of DA are discussed below.

*vs. Seg+EV*: The Seg+EV-based planner^10^ tends to prioritize path efficiency over safety because its EV-based planning costs ignore prediction uncertainties. While this tendency results in the lowest $T_\text {total}$ for all subsets but UGA, our approach maintains a comparably low $T_\text {total}$ with much higher safety for the GA and UGA subsets. These benefits stem from our time-based cost evaluation using uncertainty-aware slip estimation in (8), which balances traversability and travel time and is beneficial for successful planetary explorations that require both safety and efficiency.

*vs. MoE+CVaR*: Although the MoE+CVaR-based planner^14^ also incorporates uncertainties in slip predictions for risk-aware planning, it experiences low success rates for the UA and the most challenging UGA subsets. We found that the darker shaded regions in these subsets often cause misclassifications by the terrain classifier in the MoE slip model. Conversely, our DPT fuses color-based and geometry-based features to predict slip distributions end to end, effectively disambiguating terrain colors and shading effects to improve predictions.

**Table 2:** Domain adaptation effects for traversability prediction.

	In-domain	UG	UA	UGA
Before DA	8.7 ± 1.6	12.3 ± 2.6	12.6 ± 1.2	16.3 ± 2.3
After DA	8.7 ± 1.5	11.7 ± 1.8	11.1 ± 1.3	14.3 ± 2.1

Mean absolute errors of traversability predictions [%] by the proposed DPT model before and after the domain adaptation.

*Impact of domain adaptation*: DA successfully improves the success rates and $s_\text {max}$ scores for the UA and the most challenging UGA subsets. For the in-domain subset, the DA maintains the same performance as before adaptation, as the new in-situ samples fall within the pretraining domain. However, for the UG subset, we observe a slight decrease in the success rate. This subset poses the difficulty of adapting and correctly extrapolating the learned slip functions to OOD slope angles (*i.e.*, $|\phi |> 17^\circ$) given a few OOD samples. Because the current architecture of our slip model does not strictly follow the monotonicity inherent in latent slip functions $s = f(\phi )$, such an extrapolation through few-shot adaptation may be challenging. Incorporating monotonicity into the DPT slip model may improve its performance and adaptation stability. Still, we found that DA consistently improves the average slip prediction accuracy across the four test subsets. The results are provided in Table 2, comparing the mean absolute errors of the slip predictions for entire map regions before and after test-time adaptation.

**Fig. 3:**
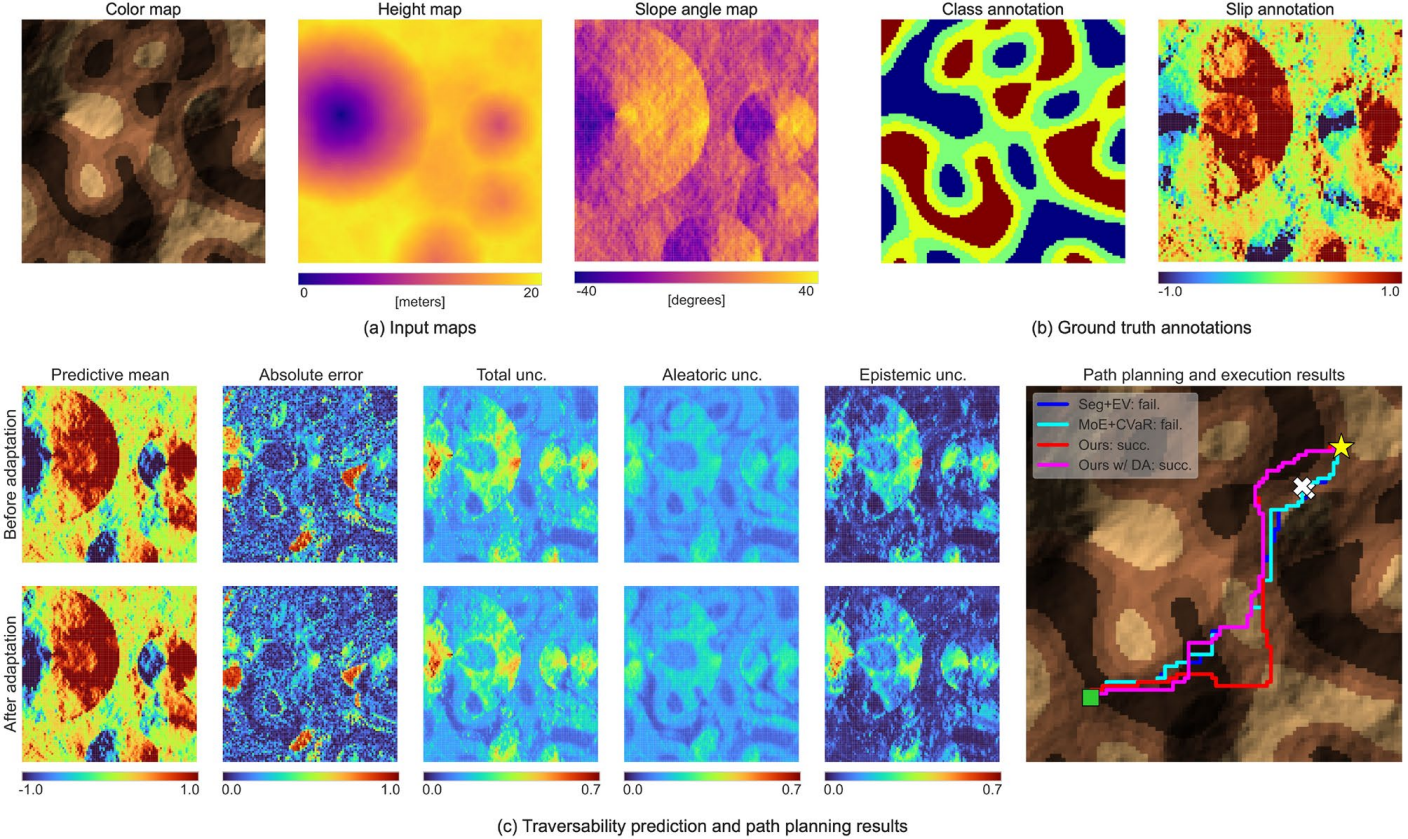
Rover navigation using the proposed unified framework. Results are compared with methods^10,14^ for an unfamiliar geometry and appearance subset problem instance. (**a**) Color, height, and slope maps as model inputs for rightward movements. (**b**) Ground truth annotations, with class annotations used for training terrain classifiers, while DPT only requires slip annotations. (**c**) Traversability predictions and path planning results. Left: Slip predictions before (top row) and after (bottom row) domain adaptation, displaying absolute errors with total, aleatoric, and epistemic uncertainties. Right: Path planning and execution results. The green square and yellow star indicate the start and goal locations, respectively. White crosses mark locations where rovers failed to traverse ($|s|=1$).

**Fig. 4:**
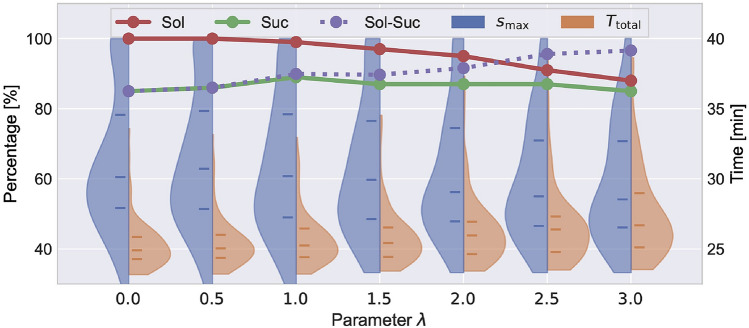
Parameter study for the unfamiliar geometry and appearance subset. Results show the variations of five metrics with varying $\lambda$. The left percentile y-axis represents Sol, Suc, Sol-Suc, and $s_{\text {max}}$, while the right y-axis represents $T_{\text {total}}$. Blue and orange violin plots represent the distribution of $s_{\text {max}}$ and $T_{\text {total}}$, respectively, with lines marking quartiles.

#### Qualitative discussion

Figure 3 summarizes rover navigation results using the proposed unified framework in a UGA subset problem instance, including input maps for traversability prediction (Fig. 3a), along with ground truth annotations for model training (Fig. 3b). Figure 3c shows the probabilistic slip predictions for rightward movements, comparing the results before and after DA.

In the results of the pre-trained DPT model before adaptation (top row), the absolute error map shows robust slip predictions, except in OOD terrain areas, such as crater-type terrain with steep inclinations and intense shading. The errors in these areas are highly correlated with the intense uncertainties in the total uncertainty map. This capability provides a comprehensive assessment of the predictions and their confidence, helping to identify both hazardous and unfamiliar terrain conditions. Uncertainty decomposition further reveals epistemic components around OOD areas and aleatoric components aligned with terrain class patterns owing to class-dependent noise in the latent slip functions.

In the results of the adapted DPT model (bottom row), we observe reduced errors for OOD regions while maintaining the overall prediction trends. This robust adaptation is enabled by our adaptation model, which tunes only the scale and bias parameters.

On the right side of Fig. 3, we compare the planned paths obtained using our method and baseline methods. Our method achieves more robust path planning than the other methods by integrating these well-calibrated uncertainty estimates into traversability evaluation. The MoE+CVaR-based planner also exploits this uncertainty. However, its misallocated class probabilities cause the same traverse failure as that caused by the Seg+EV-based planner.

#### Parameter study

We assess the impact of uncertainty exploitation for the UGA subset by running our algorithm using $\lambda \in \{0.0, 0.5, \ldots , 3.0\}$ without DA. We introduce additional performance metrics: *solved rate* (Sol) [%] as the percentage of problem instances for which a planner finds solutions ($100 \times N_{\textrm{solved}}/N_{\textrm{total}}$) and *solved success rate* (Sol-Suc) [%] as the success rate within solved cases ($100 \times N_{\textrm{success}}/N_{\textrm{solved}}$). Figure 4 illustrates how $\lambda$ controls the balance between safety and efficiency: higher $\lambda$ values favor less slippery yet longer paths, while lower values allow aggressive maneuvers to reduce travel time on risky paths. Higher $\lambda$ also reduces solved rates as inflated costs in uncertain regions block paths to the goal. For safer navigation, a higher $\lambda$ is preferable. Although increasing $\lambda$ results in lower solution rates, it improves solved success rates, suggesting that planning failures effectively predict serious immobilization risks in the environment. The optimal $\lambda$ depends on the mission specifics and environment, but statistical considerations offer general guidelines. Accounting for prediction errors within the 2$\sigma$ range (95% confidence interval) using $\lambda =2$ balances the safety and efficiency across various scenarios.

#### Open issues and possible extensions

Further refinement of traversability modeling and uncertainty exploitation can improve risk-aware planning. Potential improvements include the adoption of monotonic neural networks for slip modeling^54^ and statistical theories, such as CVaR, for risk assessment. We can also explore active learning strategies^33^ using our framework by leveraging the epistemic uncertainty components of the DPT as cues for regions that require more training samples. However, ensuring the safety of rovers during such risk-taking behaviors remains critical. Beyond these algorithmic improvements, validation for actual deployment is essential, as our simulation does not fully capture the complexities of real terrain interaction.

## Conclusion

We have introduced a unified learning and planning framework for navigating rovers on deformable terrain in planetary environments, focusing on ML-based traversability prediction and uncertainty handling. To test the effectiveness of our method, we created a synthetic dataset that highlights the challenge of train-test domain shifts in traversability prediction. Extensive simulation experiments using this dataset have demonstrated that our approach enables safe rover traverse with improved success rates compared to existing approaches, even under unfamiliar geometry and appearance conditions. Test-time domain adaptation through the end-to-end architecture of our ML model also contributes to successful navigation by continuously reducing prediction errors as the rovers traverse. Future work will explore active learning to improve domain adaptation efficiency. By leveraging epistemic uncertainty for data collection, the adaptation process could be accelerated in novel environments. In addition, we plan to incorporate real-world data (e.g., the Martian terrain data^55^) to examine scenarios with unfamiliar environmental features, analogous to those in our unfamiliar geometry and appearance dataset. This will further verify the effectiveness of our approach and highlight the thoroughness of the datasets prepared in this study.

## Electronic Supplementary Material

Below is the link to the electronic supplementary material.

Supplementary Material 1

## Data Availability

The synthetic datasets and source code created for this study are available from the corresponding author on reasonable request.
