# Supplementary material for: Deep probabilistic traversability with test-time adaptation for uncertainty-aware planetary rover navigation
Source: Sci Rep. 2026 Feb 18;16:9499. doi: 10.1038/s41598-026-40109-1 (PMC13004946; doi:10.1038/s41598-026-40109-1)
Supplement: Supplementary file 1 — Supplementary Material 1 [file 41598_2026_40109_MOESM1_ESM.pdf]

Supplementary Information for: Deep Probabilistic Traversability with Test-time Adaptation for Uncertainty-aware Planetary Rover Navigation

Masafumi Endo[1,*], Tatsunori Taniai[2], and Genya Ishigami[1]

[1]Space Robotics Group, Department of Mechanical Engineering, Keio University, Yokohama 223-8522, Japan
[2]OMRON SINIC X Corporation, Tokyo 113-0033, Japan
[*]masafumi.endo@keio.jp

ABSTRACT

This document provides supplementary information for the main manuscript.

Additional Navigation Results Across Test Subsets

These figures provide additional visual results to complement Fig. 3 in the main manuscript, which shows a single problem instance from the unfamiliar geometry and appearance subset. Here, we present four representative instances for each of the four test subsets to show how our approach performs across different conditions. Each row displays color maps as input (left) and path planning results (right). The green square and yellow star indicate the start and goal locations, respectively. White crosses mark locations where rovers failed to traverse ($|s| = 1$).

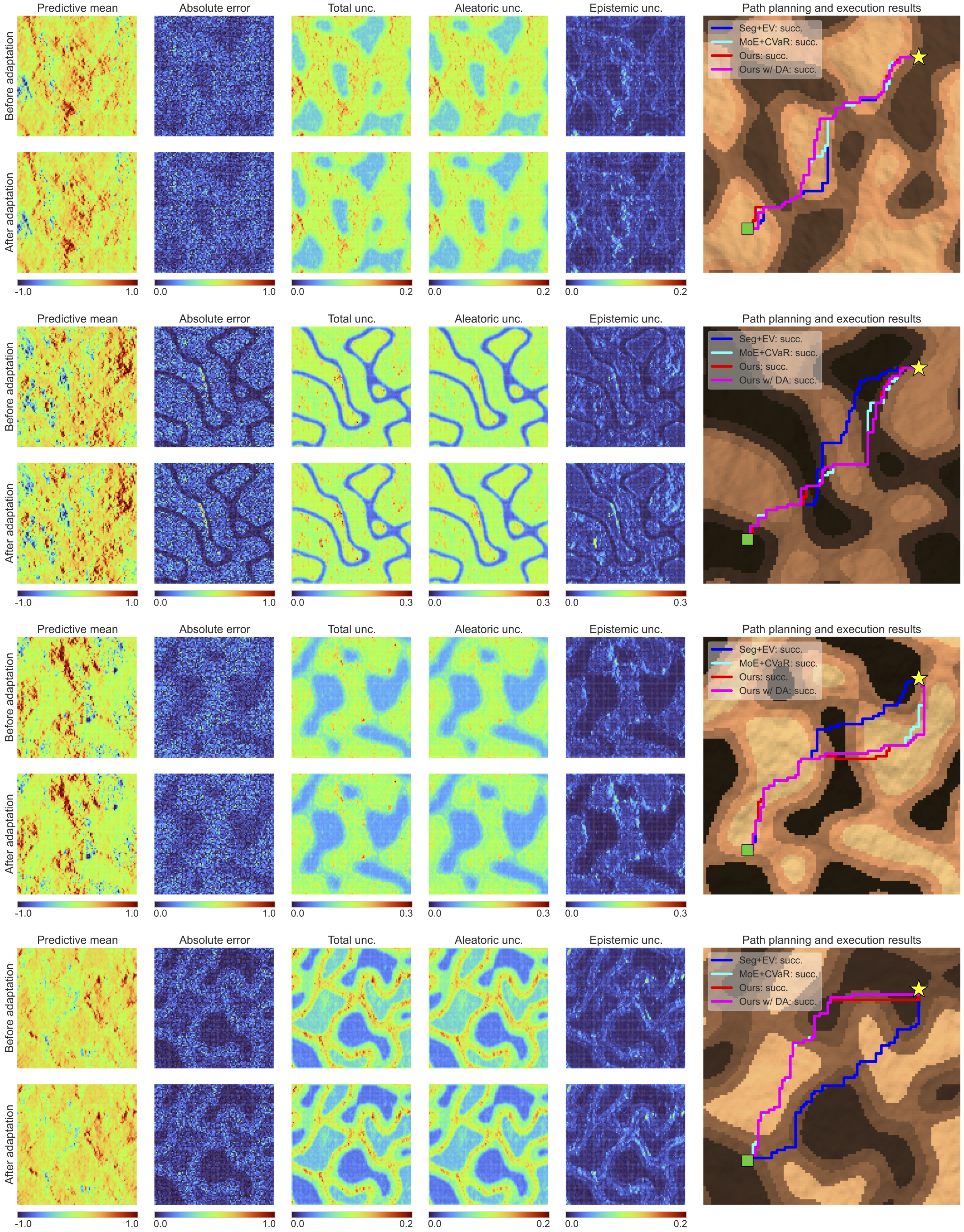

Figure 1. Additional navigation results for the in-domain subset. Four representative problem instances showing path planning and execution results.

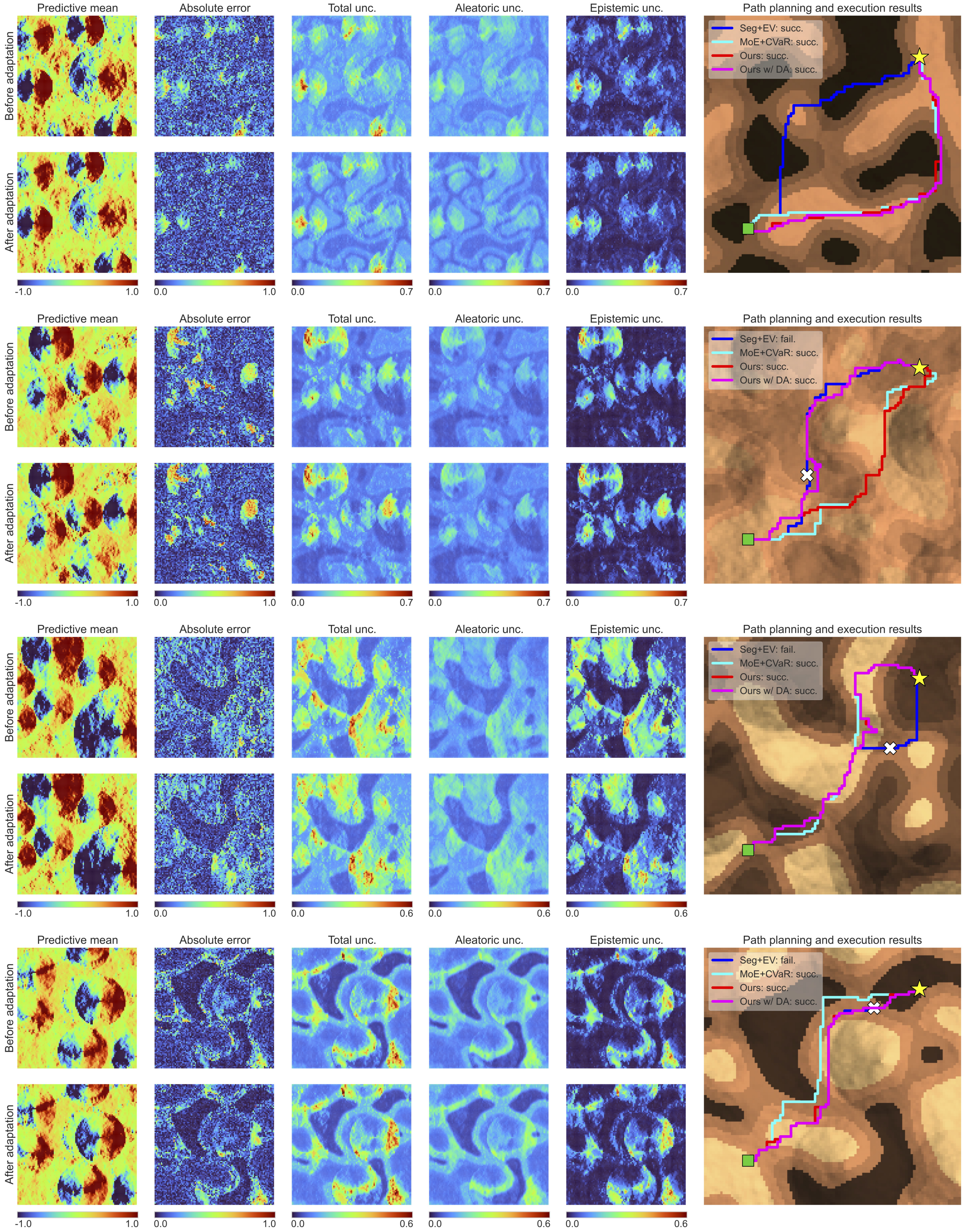

Figure 2. Additional navigation results for the unfamiliar geometry (UG) subset. Four representative problem instances under OOD crater-like geometries with steeper slopes.

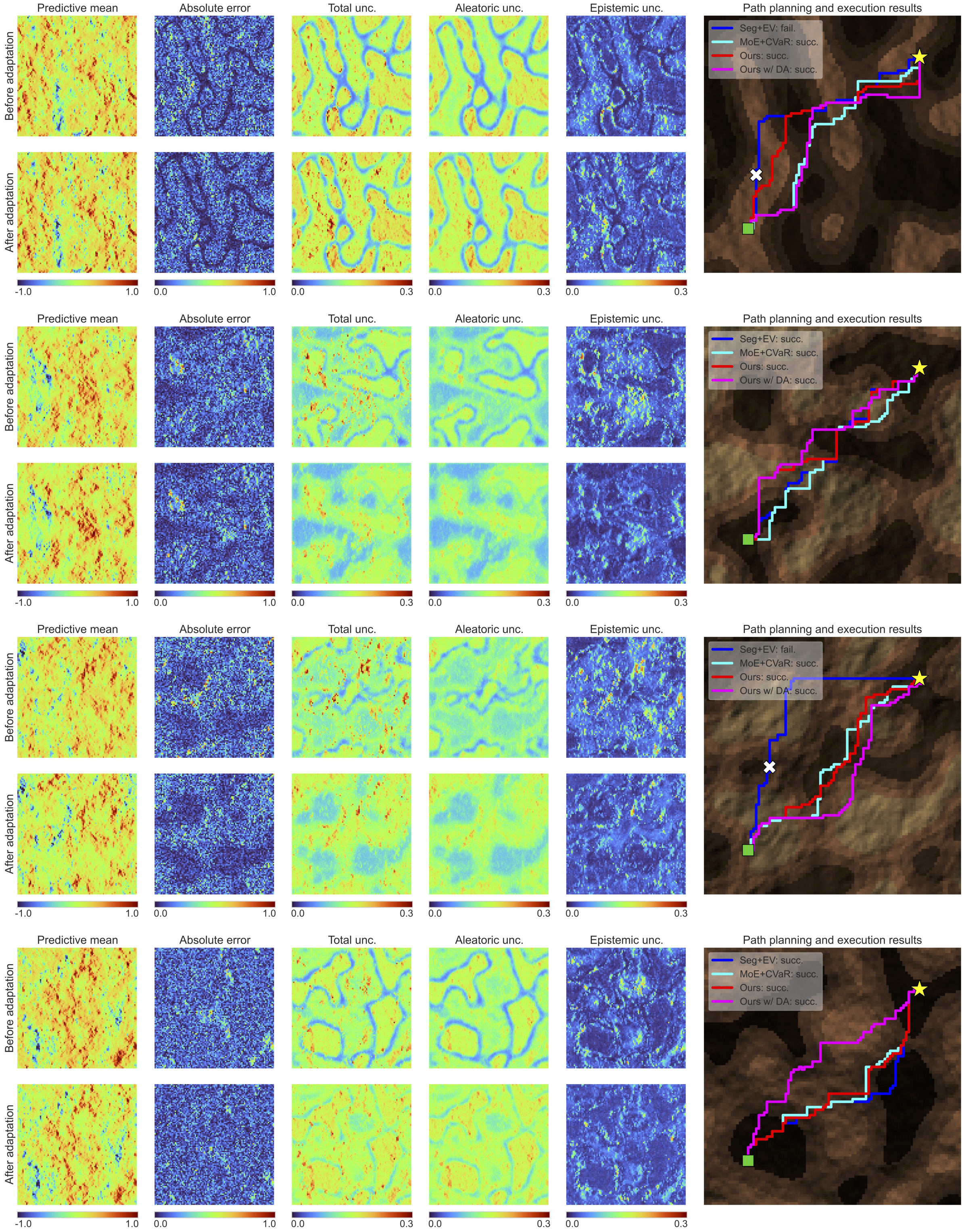

Figure 3. Additional navigation results for the unfamiliar appearance (UA) subset. Four representative problem instances under OOD appearances with darker shading.

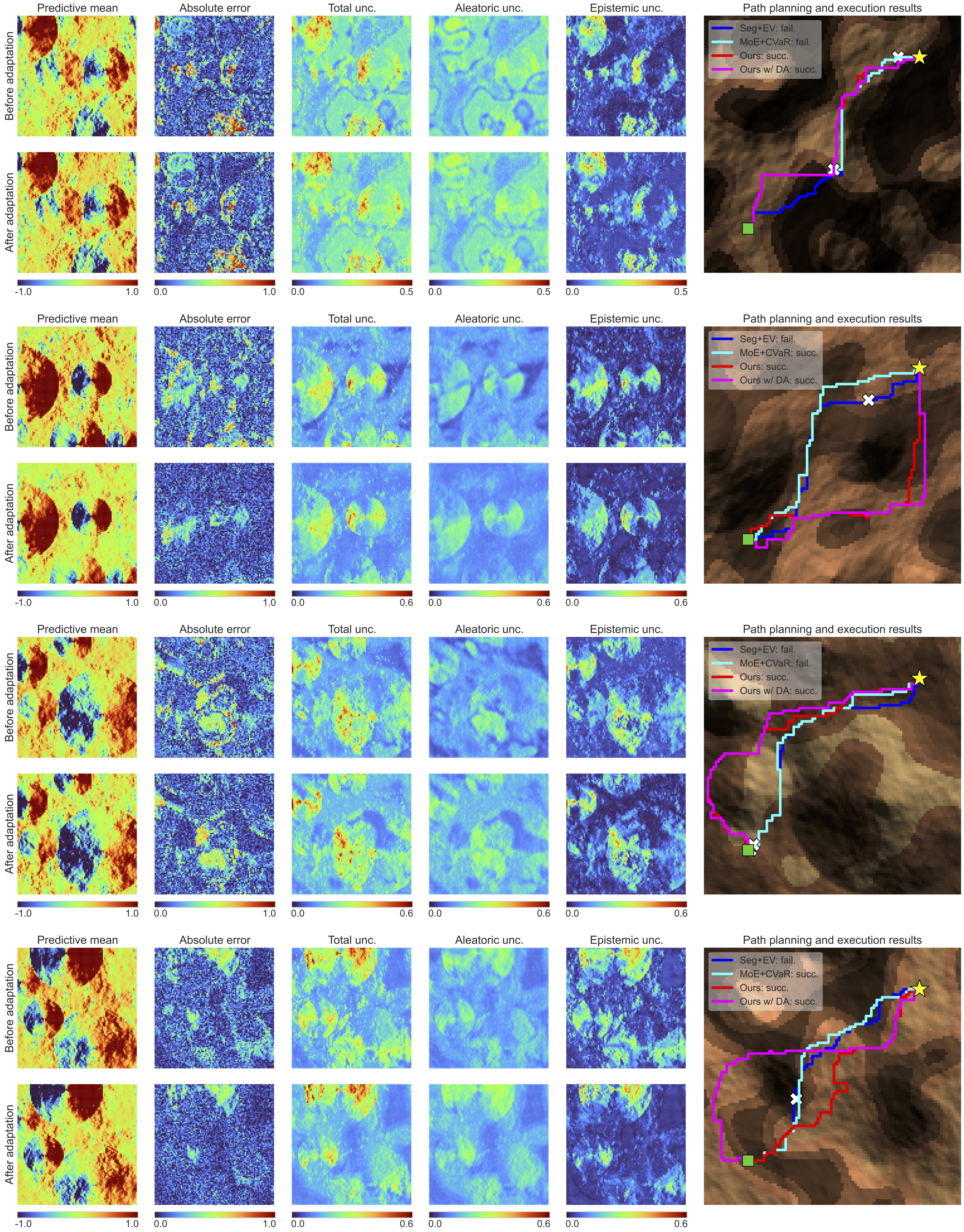

Figure 4. Additional navigation results for the unfamiliar geometry and appearance (UGA) subset. Four representative problem instances under combined OOD conditions.
